# Managing oral health care and prevention: The experience of Aboriginal and Torres Strait Islanders living in a rural community in Queensland, Australia

**DOI:** 10.1111/ajr.12853

**Published:** 2022-02-23

**Authors:** Anna Tynan, David Walker, Taygan Tucker, Barry Fisher, Tarita Fisher

**Affiliations:** ^1^ Baillie Henderson Hospital Darling Downs Health Toowoomba Queensland Australia; ^2^ The Rural Clinical School The University of Queensland Toowoomba Queensland Australia; ^3^ School of Dentistry Faculty of Medicine and Health University of Sydney Camperdown New South Wales Australia; ^4^ Kingaroy Hospital Darling Downs Health Kingaroy Queensland Australia; ^5^ Darling Downs Health Murgon Queensland Australia; ^6^ Present address: Oral Health Services – Nhulunbuy Top End Health Services Northern Territory Government Endeavour Square Gove Dental Clinic Nhulunbuy Northern Territory Australia

**Keywords:** Australia, Indigenous, oral health, rural health, treatment seeking behaviour

## Abstract

**Objective:**

To understand the experience of rural Aboriginal and Torres Strait Islanders in engaging with oral health care services and programs in order to support the development of oral health services and prevention programs that better meet their needs.

**Design:**

The study used a qualitative research design, which aims to describe participants' lived experience of engaging with oral health services and prevention programs in a rural Aboriginal and Torres Strait Islander community. Focus group discussions and in‐depth interviews were conducted with 27 participants. The 15 transcribed discussions were analysed using a 6‐step phenomenological process.

**Setting:**

A rural community in Queensland, Australia, with a predominantly Aboriginal population.

**Participants:**

Participants were purposively recruited from established health and community groups.

**Main outcome:**

System‐level barriers to accessing and engaging with oral health services and prevention influence how communities manage oral health and seek treatment.

**Results:**

The study identified 4 main themes describing the community's experience: service location and the efforts required to access oral health services; the financial burden of accessing oral health care and practising prevention; lack of confidence in oral health services; and the avoidance or delaying of accessing care for dental problems. Results confirmed a high burden of oral disease but limited attendance at an oral health facility and difficulties engaging in preventative oral health behaviours. Treatment seeking was usually instigated by the experience of pain and typically at a tertiary health facility.

**Conclusion:**

Aboriginal and Torres Strait Islanders in rural communities experience a high burden of oral disease but have limited engagement with oral health services. This is associated with system‐level barriers to accessing and engaging with oral health services and prevention.

**What is already known about the subject:**

- Aboriginal and Torres Strait Islanders have higher rates of dental caries, periodontal disease, tooth extractions and missing teeth than non‐Indigenous Australians
- Aboriginal and Torres Strait Islanders have lower attendance rates at oral health services and are more likely to visit with a problem then for a check‐up
- Rural and remote Aboriginal and Torres Strait Islander communities face further challenges due to limited availability of oral health services, and workforce issues including shortages of dental personnel in these settings, high staff turnover and an associated lack of cultural capability training within the dental workforce

**What this study adds:**

- Health system barriers influence how oral health services are accessed and how oral disease is managed by rural Aboriginal and Torres Strait Islander communities
- Unintended consequences of health system barriers include delaying treatment or presenting at emergency departments of hospitals for symptom relief
- This insight provides further confirmation of the need for a collaborative approach between communities, tertiary health services and oral health services in the design of programs to improved access to oral health care and prevention, and address oral health disparities particularly for rural Aboriginal and Torres Strait Islander communities

## 1 INTRODUCTION

Oral health impacts on everyday activities such as eating, speaking and social interactions and is also linked to cardiovascular disease, poor nutrition, diabetes and chronic kidney disease.^1^, ^2^, ^3^, ^4^, ^5^ In Australia, Aboriginal and Torres Strait Islanders continue to have a higher burden of oral ill‐health including dental caries, periodontal disease, tooth extractions and tooth loss compared to non‐Indigenous Australians.^6^, ^7^, ^8^ Aboriginal and Torres Strait Islanders have lower attendance at dental services; are more likely to visit for a problem than for a check‐up; are more likely to be hospitalised for oral conditions; and have poorer oral health‐related quality of life than non‐Indigenous Australians.^9^, ^10^, ^11^, ^12^ In addition, Aboriginal and Torres Strait Islander people have a higher overall disease burden, which is complicated further by a higher prevalence of social concerns. These might include low socioeconomic status; low social capital; and a higher prevalence of lifestyle risk factors such as high levels of smoking, alcohol consumption, diets high in sugar and reduced engagement in oral hygiene activities.^13^, ^14^, ^15^, ^16^, ^17^ However, despite evidence of reduced uptake of oral health care and engagement in prevention activities, there is evidence that having good oral health is an important priority for Aboriginal and Torres Strait Islander communities.^18^

Health system barriers such as affordability, accessibility and approachability are known to influence oral health outcomes, particularly for vulnerable populations.^19^ For Aboriginal and Torres Strait Islanders, system‐level barriers to accessing oral health services include difficulties accessing culturally appropriate oral health care and promotion; limited coordinated service provisions between oral health and other health professionals; high dental service cost including direct and indirect costs; and difficulties accessing transport to services.^20^, ^21^ Rural and remote communities face further challenges due to geographic locations of oral health services and the long distances community members need to travel to access care impeding regular attendance for non‐problem‐based reasons. Rural and remote communities also face health workforce issues including lack of appropriate cultural competency training of non‐Indigenous workers, staff shortages and high staff turnover.^22^, ^23^, ^24^ This in turn influences relationship and partnership building with the community and the ability of the local community to access preventative oral health treatment as most rely on regularly available and professionally trained personnel to provide interventions such as fluoride varnish.^25^, ^26^

Individual factors such as overall health, social environment, life experiences, socioeconomic status, age and gender shape how oral health is experienced and how individuals engage with oral disease prevention.^27^, ^28^ For Aboriginal and Torres Strait Islanders, individual factors might also include experiences such as institutionalised racism within health care services and lack of familiarity with dental care providers due to staff turnover.^29^, ^30^ Living arrangements and limited finances also influence access to healthy diets and oral hygiene supplies and healthy food.^31^, ^32^ Evidence also exists of poor oral health literacy among Aboriginal and Torres Strait Islander people, which has been reported as a significant risk indicator for poor self‐reported oral health and attendance for dental treatment only when experiencing symptomatic conditions.^21^, ^33^ Previous research has also shown that having no oral health impairment or low levels of periodontal disease resulted in significantly reduced attendance at an oral health service by Aboriginal and Torres Strait Islanders.^34^ Research also indicates that some Aboriginal and Torres Strait Islander people believe that it is inevitable they will have poor oral health.^35^

The purpose of this paper is to explore the experience of engaging in oral health care and prevention in a rural Aboriginal and Torres Strait Islander community and the implications this has on treatment seeking and preventative behaviour. This is considered within the social, cultural and historic contexts of Aboriginal and Torres Strait Islanders, which includes the recognition of institutionalised racism and the impact of colonisation on Aboriginal and Torres Strait Islander peoples' ability to achieve health.^29^, ^30^, ^36^, ^37^ It is anticipated that the findings will assist in deepening the understanding of oral health care and prevention experience and support the development of oral health services which better meet the needs of rural Aboriginal and Torres Strait Islander communities.

## 2 METHOD

### 2.1 Study design

This study is one aspect of a larger qualitative study exploring the community's perceptions of: the importance of oral health; oral health service experience and needs; and of how oral health can be best improved within a rural Aboriginal and Torres Strait Islander community. Improving oral health was identified as a key concern by the community and Elders through the local Health Action Group. The Health Action Group includes representatives from the diverse agencies in the community including health, education and welfare agencies. The research team include Indigenous and non‐Indigenous personnel, community and external personnel, dental and non‐dental personnel.

A qualitative research design was employed to describe, understand and interpret experience as it is lived by people in order to provide an account of the phenomena under examination.^38^, ^39^ The research foci for this paper explores the lived experience of engaging in oral health care and prevention within a rural Aboriginal and Torres Strait Islander community. This theme was derived as part of the broader research project and noted by the investigator team as an important theme in the data to undertake closer analysis. Data collection took place from February 2017 to July 2017.

### 2.2 Study setting

The study was completed in a rural community in Queensland with a predominantly Aboriginal population. At the time of the study, existing oral health services were intermittent and dominated by emergency care with the closest private dental service located approximately 8 km from the community and the closest public dental service was located approximately 50 km from the community.

### 2.3 Participant recruitment and data collection

Purposive sampling was used to recruit participants from known, established health and community groups within the community following advice from key informants. Data collection methods included individual semi‐structured in‐depth interviews (IDI) and focus group discussions (FGD) with community members and community leaders. Participants were offered the choice between an IDI and a focus group. Focus groups were organised into homogenous groupings based on feedback from participants to facilitate interaction and quality output as well as ensure participants felt comfortable in the discussions. A total of 10 male and 17 female participants were recruited ranging from 18 to over 50 years.

The initial design of questions for the interviews was developed through discussions within the research team, review of the literature and consultation with local community members to ensure cultural appropriateness and clarity of questions. The interview questions were directed to support participants to recall experience of accessing oral health services and prevention programs; managing oral health and prevention of oral disease within the community; and their experience of barriers and enablers to accessing oral health care and prevention at both the individual level and system level (Appendix 1). Questions were piloted by the team with feedback from the local team members. Discussions were led by Indigenous (TF, BF) and non‐Indigenous researchers (TT) and a local Indigenous research assistant employed under the research program with the support of 2 other members of the research team who were both experienced qualitative researchers. The Indigenous researchers and the research assistant were also community members. The non‐Indigenous discussion leader had an established relationship with the community due to her role as a dental therapist there. During the interviews, participants were encouraged to share their stories and develop knowledge in a way that was cooperative and respectful. To allow for reflexivity of the research process, the interview schedule was further refined and reviewed during the data collection period. The discussions ranged from 30 to 45 minutes and were completed at community venues suggested by participants. Field notes were also completed to assist with recording investigator observations and reflections.

### 2.4 Data analysis

All IDIs and focus groups were digitally recorded and transcribed verbatim. Participants' descriptions of their experience were studied by the researchers throughout the analysis procedure. The analysis procedure chosen for this study was based on the method developed by Colaizzi for phenomenological research.^39^ Data analysis initially involved 2 of the researchers carefully reading the transcriptions independently to gain a greater understanding of participants' experiences. During this process, significant statements were extracted from each transcript by each researcher. The underlying meaning of each significant statement was formulated using inductive reasoning. The formulated meanings were then organised into clusters of themes by each researcher. The 2 researchers then compared the themes, discussed similarities and differences and checked against original transcripts for validation until consensus was reached and data saturation confirmed. Themes were then confirmed by fellow authors and checked with the Health Action Group to ensure appropriate cultural understanding and further explore interpretation where necessary. NVivo © 12 (Windows) QRS was used to organise data.

### 2.5 Ethics approval

The study was granted ethics approval from the Darling Downs Health Human Research Ethics Committee (Protocol HREC/16/QTDD/42) and was conducted within the guidelines for Ethical Research in Australian Indigenous Studies.^40^ The research team undertook significant consultation with the community in developing and implementing the research. The research was identified as a high priority project and endorsed by the local Health Action Group. Two of the researchers were community members, and a local research assistant was employed to provide additional support in relation to appropriate implementation of the study in the local community. This included promotion of the study in the community and assistance with cultural support of participants in accessing and participating in the interviews if needed. Written informed consent was obtained from all participants. All data were de‐identified and pseudonyms used.

## 3 RESULTS

Results from the 3 focus groups and 12 in‐depth interviews revealed both individual‐level and system‐level factors influencing the experience of oral health care services; and managing oral health and competing health issues within the community. Four main themes were identified and are outlined in the following.

### 3.1 Service location and the efforts required to access oral health services

For most of the community, respondents advised that if they needed to attend an oral health service, they would need to take a 40‐minute road trip to the nearest major town. However, there were no public transport options available. The difficulties in accessing this oral health service and the impact on oral disease is summarised by the following participant statement:

One of the barriers is that they can't get up there. Or they can't get into to town to see them. Because either they got no money, or they haven't got a car, or that because there's nothing here. We've had kids with toothaches for months. (IDI 11, Female Participant)

As noted above, issue with needing to travel to oral health services was a great concern for the community due to many not having their own cars. If an individual did not have their own transport, then family and friends might be requested either to drive community members to dental appointments or to lend their cars. For those that worked, accessing oral health services also often meant taking time out away from their employment to travel to appointments for either themselves or a family member.

So another family member has got to take them which they've got to shuffle their day around to do it….Because I do stuff like that for family too when they want to loan the car, give them a loan of the car to go (for oral health appointment) to use it. There are no other ways to get up there. (IDI 10, Male Participant)

Respondents observed that there was a local community managed bus that provided travel for health appointments including oral health; however, it had several limitations. This included how many seats were allocated for oral health per trip; rigid times for pick up and drop off; and the exclusion of children requiring a car seat. This is an important restriction to highlight as early childhood caries can occur in the very young who do need a car seat. It was also noted that oral treatment was often not satisfied by one appointment and required several visits over an extended period increasing the burden of transport requirements and its barrier to access of appropriate oral health care.

### 3.2 The financial burden of oral health care and prevention

The cost associated with oral health was a key concern for all respondents. Finance issues included the direct costs involved with care such as private dentistry service fees and the indirect costs of attending appointments including transport costs; costs associated with taking time away from employment to attend appointments for individuals and others; and costs of oral hygiene products to prevent oral disease. This participant statement highlights the impact of the cost of oral health care on her oral health,

Yep. I did have one pulled a little while ago …it cost nearly $400, so that experience has then said I can never afford that. I can never afford that, so that's why my teeth are really bad. (IDI 8, Female Participant)

Financial implications extended to the incidental costs associated with attending appointments by extended family and friends

Yep. And even just for a family that can access it, taking that time out with the kids to get over there (is another issue). (FGD 1, Female Participant)

Financial strain was also identified to influence ability to participate in preventative oral health care. For example, many respondents commented on the cost of toothbrushes and toothpaste. Respondents advised that key issues in preventing oral disease also stemmed from the financial burden of accessing healthy food and the social environment of many households in the community.

You've got three or four generations, sometimes it may be three or four brothers, and that, living at home with mum and they all have their food in the cupboards; there's a lot more processed stuff. Cordial is an easier option because you can keep that in your room …. So, some have a fridge, not everyone has a fridge in their bedroom for their food, so nutrition then effects now dental…Different if they've got their own house; it's one fridge, but if you're sharing a house with 12 people you don't want to be the one buying the milk or all that good stuff. (FGD 1, Female Participant)

### 3.3 Confidence in oral health services

Community members' experiences influenced perceptions that oral health services were ‘too hard to access,’ ‘too expensive’ or have ‘too long a waiting list.’ Many reported hesitations in accessing services and lengthy delays in getting care. For a few respondents, experiences of difficulty with attempting to make appointments also affected confidence in the system and feelings of unreliability of the service. These issues are illustrated in the following comment regarding trying to access a service locally.

I put my name on the list and we just wait till they come through. That was postponed twice, I think it was…because they couldn't come through. So they changed it from one month to the next and then they couldn't get (someone) in so they had to wait another ‐ well, we had to wait another month until they came through… Well, my daughter was in a lot of pain. We couldn't afford for her to go into town to the private dentist, so I guess it's hard when you are waiting for them to come through and I'm not sure if it's going to be on the date that they say, and just waiting. (IDI 6, Male Participant)

Respondents also raised concerns about contacting oral health services to arrange appointments.

So, I did try ‐ there was a van here a couple of years ago, but there was a number to call but there was no response whatsoever (for local dental van). So how do you get help with your teeth when there's no response from anyone? (IDI 12, Female Participant)

The issue of waiting lists was raised by many of the respondents, expressing there was an expectation of a very long wait for service. There was concern among participants that waiting lists in the public system could be years long depending on the urgency of the need.

My husband, he's got gum disease ‐ his teeth are all loosened, and so he needs to have them removed. He needs false teeth. We're having major issues trying to find help for that. If he was to wait at the public dentist it's up to ‐ I think it was a four to five year wait for him to even be looked at for false teeth. (IDI 8, Female Participant)

As a result of a history of intermittent local services, particularly for adults, many felt that oral health service provision in the community was unreliable.

… services have come and gone; they're light, hey? They're unreliable. They're not consistent. (FGD 1, Female Participant)

A few older respondents also discussed a general reluctance to access any health service due to previous experience of racism within health services.

Some of the older, our age. They don't like coming to this building (a health facility) simply because it was a no‐go zone. It was known as white only building (during the days of the mission). (IDI 7, Female participant)

### 3.4 Avoiding or delaying care for a tooth problem

Nearly all respondents described experience with avoiding or delaying access for oral health complaints. Seeking professional treatment was reported to usually only occur when the problem caused very severe pain, with many participants describing experiencing extreme dental symptoms while delaying treatment.

I remember I did too (put off seeing a dentist) with one of my teeth. I was putting that off for a long time and it was excruciating man! I lay in the dark and just put a hot rag on my face [for] the pain. (IDI 10, Male Participant)

A few participants discussed fear of dental care, including a fear of dental needles and a fear of dental treatment pain, as a reason for not accessing an oral health service. Participants stated that the social stigma of having bad teeth might also result in delaying treatment seeking due to the disclosure of the unhealthy condition of their mouth. However, for the majority, experience with delaying care for a tooth problem was due to concerns about cost, difficulties with accessing services and the existence of multiple, competing priorities.

I've got no money so I'm just going to suffer it until I do. But when you do come to get it, they come around to do it, your side‐tracked with everything else. You say, ‘I've got to pay this bill first,’ then ‘I've got to pay that bill’ and you say to yourself ‘Oh, I got to definitely do it this week!’ (FGD 2, Male Participant)

In some cases, concerns about cost meant community members sought alternative ways of managing their oral health complaint, particularly when the discomfort became unbearable. This included participants pulling out their own teeth and being asked to pull out other community member's teeth.

I've known people ‐ they've just come over with pliers and said can you pull this out for me, because they can't afford to go to the dentist. (IDI 8, Female Participant)

A small number also advised of using alcohol so that they could control the pain of pulling out their own teeth.

Well I've got no car now and just before Christmas yeah, we were at a breaking up party here and that night, you know my tooth was aching, yeah, so what I do yeah, I just get drunk and just pull it out. (FGD 2, Male participant)

For the majority of respondents, the most commonly reported way to manage their oral health concerns was attending the emergency department at the local hospital.

Me, I haven't been to the dentist for years. Shocking hey? I normally just go to hospital. (IDI 7, Female Participant)

I put up with the pain. Until it gets real bad then I go to hospital and stress out on them. (IDI 3, Female Participant)

## 4 DISCUSSION

This study explores the experience of a rural Aboriginal and Torres Strait Islander community in engaging in oral health care and prevention and the implications this has on treatment seeking and preventative behaviour. It identifies several system‐level barriers to accessing and engaging with oral health services and prevention that influence how the community manages oral health and seeks treatment. In particular, it shows that health system barriers such as cost of care and prevention, location of services, availability of transport options and confidence in the health system influences how oral health services are accessed and how oral disease is managed by the community.

The study shows that community members are managing the difficulties within the health system the best way they can; however, this has resulted in unintended consequences. For example, ongoing unmanageable symptoms such as tooth pain prompted treatment seeking behaviour at emergency departments of hospitals or self‐administration of own treatment such as pulling teeth. In particular, the study highlights treatment being delayed and often not sought at an oral health service due to the difficulties and unreliability of access to oral health care. This study confirms that system‐level barriers play a key role in treatment seeking behaviours of rural Aboriginal and Torres Strait Islanders and potentially lead to attendance at hospitals for dental disease treatment. Further research is needed into the economic impact of preventable oral disease from being managed by health service emergency departments.

The study also indicates that individual factors effect engagement with oral health services and prevention activities. Barriers such as poverty have multiple impacts on engaging with oral health services and the adoption of preventative behaviours in this community. Limited access to finance influences a person's ability to own a car, organise travel to attend an oral health service and to purchase preventative products.

It is likely that a collaborative approach between health services and communities in oral health service design could lead to improved access to services and oral health outcomes. Oral health services and promotion programs for Aboriginal and Torres Strait Islanders should be developed in consultation with communities and designed to meet the needs and challenges of specific communities.^41^, ^42^ Research has shown that oral health services targeted towards Aboriginal and Torres Strait Islanders are more likely to be effective if the strategy addresses the social determinants of health, is sustainable and is owned by the community.^36^ There is a need to formalise these relationships in oral health service planning documents and policies which allow for the evaluation of the extent to which communication has been conducted by health services with local communities. Along with this, there is a need for collaborative research to learn how to best develop these consultative relationships. However, it is noted that improving access to service alone will not solve the problem of poor oral health outcomes in rural Aboriginal communities. There is also need for a multi‐sector approach that recognises the importance of access to healthy food options, oral hygiene supplies and oral health messaging. Further research into understanding how this might be done from the perspectives of Aboriginal and Torres Strait Islander communities is needed.

## 5 CONCLUSION

Aboriginal and Torres Strait Islander in rural communities have a high burden of oral disease but limited engagement in oral health services. This highlights the need to develop oral health services which meet the needs of rural Aboriginal and Torres Strait Islander communities. Success in meeting these needs is likely to be the result of multiple strategies selected in partnerships with local communities who best know their oral health needs and the barriers they face in accessing care; and not restricted to the development and delivery of oral health services in isolation from the community.

## CONFLICT OF INTEREST

The authors declare that they have no competing interests.

## AUTHOR CONTRIBUTIONS

AT: conceptualization; data curation; formal analysis; funding acquisition; methodology; supervision; writing – original draft; writing – review & editing. DW: conceptualization; formal analysis; methodology; supervision; writing – review & editing. TT: conceptualization; data curation; funding acquisition; investigation; project administration; validation; writing – review & editing. BF: investigation; methodology; project administration; resources; writing – review & editing. TF: conceptualization; formal analysis; methodology; project administration; resources; supervision; writing – review & editing.

## DISCLOSURE

This research was funded through the Department of Health—Health Practitioner Research Scheme 2016/2017 funding round. The funding body had no role in study design, data collection and analysis or writing of the manuscript.

## APPENDIX 1

### Interview Guide for Focus group discussions and In‐depth interviews with community members

The theme list did not rigidly structure the discussions but instead was used as a guide to the discussion where necessary.

### Theme: Experience of managing oral health as a community member

- Describe what it has been like to look after your teeth? And your family's teeth?
- How have you managed to look after your teeth? And your family's teeth?

Prompts:

- Can you give me an example?
- Why do you think this?

### Theme: Barriers and facilitators to managing oral health in the community

- Described how people look after their teeth and mouths in your community?
  - What do they do?
  - What has helped?
  - What has been difficult?
  - Can you give examples?

### Theme: Perception of oral health care needs for the community

- What are the needs for looking after your teeth here in community?
- Who should be targeted and how?
- What resources are available here?
- What services exits?
- What is missing?
